# Extracellular matrix in ascending aortic aneurysms and dissections – What we learn from decellularization and scanning electron microscopy

**DOI:** 10.1371/journal.pone.0213794

**Published:** 2019-03-18

**Authors:** Teresa Mimler, Clemens Nebert, Eva Eichmair, Birgitta Winter, Thomas Aschacher, Marie-Elisabeth Stelzmueller, Martin Andreas, Marek Ehrlich, Guenther Laufer, Barbara Messner

**Affiliations:** 1 Department of Surgery, Cardiac Surgery Research Laboratory, Medical University of Vienna, Vienna, Austria; 2 Department of Surgery, Cardiac Surgery, Medical University of Vienna, Vienna, Austria; Michigan Technological University, UNITED STATES

## Abstract

Pathological impairment of elastic fiber and other extracellular matrix (ECM) components are described for the aortic media of ascending thoracic aortic aneurysms (aTAA) but the exact pathological impairment of the structure and its degree still needs further investigations. To evaluate the quantity and quality of elastic fiber sheets and other ECM structures (e.g. collagen), cells were removed from different types of aneurysmal tissues (tricuspid aortic valve [TAV] associated-, bicuspid aortic valve [BAV] associated-aneurysmal tissue and acute aortic dissections [AAD]) using 2.5% sodium hydroxide (NaOH) and compared to decellularized control aortic tissue. Likewise, native tissue has been analysed. To evaluate the 2D- (histological evaluation, fluorescence- and auto-fluorescence based staining methods) and the 3D structure (scanning electron microscopic [SEM] examination) of the medial layer we first analysed for a successful decellularization. After proving for successful decellularization, we quantified the amount of elastic fiber sheets, elastin and other ECM components including collagen. Aside from clearly visible focal elastic fiber loss in TAV-aTAA tissue, decellularization resulted in reduction of elastic fiber auto-fluorescence properties, which is perhaps an indication from a disease-related qualitative impairment of elastic fibers, visible only after contact with the alkaline solution. Likewise, the loss of collagen amount in BAV-aTAA and TAV-aTAA tissue (compared to non-decellularized tissue) after contact with NaOH indicates a prior disease-associated impairment of collagen. Although the amount of ECM was not changed in type A dissection tissue, detailed electron microscopic evaluation revealed changes in ECM quality, which worsened after contact with alkaline solution but were not visible after histological analyses. Apart from the improved observation of the samples using electron microscopy, contact of aneurysmal and dissected tissue with the alkaline decellularization solution revealed potential disease related changes in ECM quality which can partly be connected to already published data, but have to be proven by further studies.

## Introduction

According to World Health Organization (WHO), thoracic aortic aneurysms (TAAs) belong to the group of cardiovascular diseases, which are the number one cause of death worldwide.[1] In 2014 in the United States, 9,863 deaths were due to an aortic aneurysm.[2] According to their location, TAAs can be divided into ascending and descending aortic aneurysms. They also show differences in their pathology affecting the proper wall function, which are suspected to be due to the different origins of the vascular smooth muscle cells (SMCs). While in the ascending part of the aorta, SMCs come from the neural crest, the SMCs from the descending aorta origin from the paraxial mesoderm.[3] Aside from these hypotheses, the underlying cause of non-syndromic ascending thoracic aortic aneurysm (aTAA) formation is completely unknown.

The aortic wall of aneurysm patients is characterized by typical medial degeneration, which is displayed by a loss of SMCs and a fragmentation of elastic fibers.[4] In tricuspid aortic valve (TAV)-aTAAs the medial degeneration is more pronounced which can be seen as a loss of fiber orientation and a decrease in smooth muscle mass compared to bicuspid aortic valve (BAV)-aTAAs. Furthermore, the number of cell nuclei in the tunica media is reduced in BAV-aTAAs compared to TAV-aTAAs.[5,6]

Of note, Tang et al.[7] showed that total collagen- and elastin protein amount are decreased in aneurysms compared to non-aneurysmal (NA) tissue although the ribonucleic acid expression of collagen I and III as well as elastin and biglycan were not altered. In addition, the degree of elastic lamellae fragmentation in the medial layer progressively rises from the internal to the external elastic lamina.[7] Likewise, also Borges et al.[8] found out that the decreased collagen content in the internal media is associated with aTAAs. Overall, less collagen can be found in aTAAs compared to normal aortic tissue [8], although also opposite results were presented [9,10].

In contrast to aneurysms, aortic dissections are characterized by the creation of a false lumen for blood flow, induced by a to date unidentified damage within the aortic media.[11,12] In contrast to aneurysms, aortic dissections often have a more fatal outcome with a higher mortality rate because many patients do not reach the hospital on time. Over a long period of time it was believed that the dissection was a consequence of an aneurysm. Now, however, it becomes more and more apparent that dissection is a separate disease from aneurysm, since the majority of dissection patients (>80%) have no aneurysm before.[13] Therefore, we used also tissue from type A dissection patients for analysis to demonstrate potential similarities and differences which might further help to characterize this disease in comparison to aneurysms. A type A dissection is according to the Stanford classification, a dissection located in the ascending aorta while with a type B dissection the descending thoracic aortic wall is affected.[14] In addition, acute aortic dissections (AADs) are described to be associated with cystic medial necrosis (e.g. reduced SMCs, fragmentation of elastin).[13] As Borges et al.[8] showed for aneurysms, they also observed an overall reduced amount of medial collagen, especially in the media adjacent to the adventitia for AADs.

In general, elastin and collagen belonging to the extracellular matrix (ECM) are important to maintain tensile strength and stiffness of the aortic tissue.[15] Elastin components in the medial layer of normal aorta can be found as elastic lamellae, which are circumferentially and parallel arranged sheets with a fibrous surface. Other parts are the interlamellar elastin fibers in between and surrounding the SMCs as well as elastin struts connecting two adjacent elastic lamellae. These elastin struts are also involved in bearing radial loads which should avoid the formation of aortic dissection or delamination.[16,17] Collagen in the medial aortic layer can be found in between elastic lamellae and also around SMCs as interspersed collagen fibers and thin bundles. However, collagen fiber bundles are parallel arranged but with different orientation so more fibers are present at a smaller area.[16] Additionally, collagen type I and III are the most abundant collagen types in the aortic tissue of all known types.[15] Therefore the aortic ECM composition plays an important role in regard to stability and overall aortic wall function and therefore also concerning aneurysm and dissection development.

In the present study we aimed at analysing the ECM of aneurysm and dissection tissues compared to corresponding controls in more detail by decellularization of tissue and conducting histological stainings, fluorescence-based analyses as well as qualitative assessments of the 3D structure using scanning electron microscopy (SEM). By using and comparing decellularized aortic tissue we hope to mainly facilitate our electron microscopic based analyses. After preliminary tests with detergents and the associated incomplete decellularization, we decided to perform the decellularization with an alkaline solution. To establish our method of decellularization we referred to the publication of Borges et al.[17] and also used sodium hydroxide (NaOH), but in a much lower concentration to prevent an overall damage to ECM components. Of note, by comparing the quantifications from analysing decellularized tissue with native tissue and aneurysmal with non-aneurysmal, we tried to distinguish and point out pathological disease-related ECM alterations (quantitative and qualitative changes) seen in native tissue with those alterations which became apparent after contact with the alkaline solution in decellularized tissue. This could help us in understanding disease-related pathological changes. In the course of the present study we focus on how the decellularization method influences the ECM quality and structure by using different imaging techniques. It is worth noting that decellularization with an alkaline solution can also cause damage to the ECM, which must not be over interpreted and therefore a comparison with the corresponding native tissue and appropriate non-disease tissue is necessary.

## Materials and methods

### Aortic tissue samples

The different types of ascending aneurysmal tissues (BAV-aTAAs, n = 9; TAV-aTAAs, n = 9; AADs type A, n = 8) were obtained from patients undergoing surgery for aneurysm or dissection development who gave their written consent for using their tissue for research after the procedure was explained in detail by the physician. Exclusion criteria were associated genetic diseases and signs of inflammation (e.g. aortitis). Control aortic tissues from organ donors or recipients were used as control group (NA; n = 9). Patients’ characteristics as sex, mean age and aortic diameter are listed in Table 1. Until further investigation, aortic tissue samples were stored in RPMI 1640 media (Lonza, Basel, Switzerland) supplemented with 10% fetal calf serum and 1% Penicillin-Streptomycin at 4°C. The study was approved by the Ethics Committee of the Medical University of Vienna (EK Nr: 1280/2015). If not stated otherwise, reagents were purchased from Sigma-Aldrich.

**Table 1:** Overview of patient group characteristics.

	Sex	Mean age ± SD	Aortic diameter [cm]	n
NA	M = 6, F = 3	51 (± 18.5) years	nd	9
BAV-aTAA	M = 6, F = 3	53 (± 12.6) years	5.33 (+/- 0.25)	9
TAV-aTAA	M = 5, F = 4	69 (± 7.5) years	5.40 (+/- 0.43)	9
AAD type A	M = 5, F = 3	67 (± 8.6) years	nd	8

NA = non-aneurysmal tissue; BAV-aTAA = bicuspid aortic valve associated ascending thoracic aortic aneurysm; TAV-aTAA = tricuspid aortic valve associated ascending thoracic aortic aneurysm; AAD type A = acute aortic dissection type A; M = male; F = female; SD = standard deviation; nd = not determined; n = number.

### Decellularization of aortic tissue using sodium hydroxide (NaOH)

All decellularization steps of the aortic tissue were performed without shaking at room temperature. Samples for decellularization were cut into pieces with a size of 1cm x 0.5cm. First, the aortic tissue was washed in phosphate buffered saline (PBS) without CaCl_2_ and MgCl_2_ for 30 minutes before incubation with 2.5% NaOH (Merck, Darmstadt, Germany) for 48 hours, adopted from Borges et al.[17]. Then, tissues were washed in PBS for 24 hours followed by incubation with 1% tannic acid for 48 hours for tissue stabilization.[18] Finally, samples were washed in PBS for 10 minutes prior to fixation in 4.5% formaldehyde (SAV Liquid production GmbH, Flintsbach, Germany) or 2.5% glutaraldehyde (Agar Scientific, Essex, United Kingdom).

### Tissue processing after decellularization

After fixation in 4.5% formaldehyde for 48 hours, paraffin embedding was performed using a KOS microwave HistoSTATION (Milestone, Sorisole, Italy) by putting samples in absolute ethanol (35 minutes), followed by isopropanol (70 minutes) and lastly paraffin (90 minutes). Tissue samples were then cut in 5μm sections for histological and fluorescence-based stainings.

### Histological stainings

Sections were deparaffinized by HistoSAV (Liquid Production GmbH, Flintsbach, Germany) incubation for 20 minutes, followed by a decreasing ethanol series (2 times absolute ethanol, 2 times 96% ethanol, 80% ethanol, 70% ethanol, 50% ethanol one minute each) until aqua dest. to rehydrate tissue sections.

Elastika van Gieson staining was performed following manufacturer’s instructions (Sigma HT-25). In short, after deparaffinization, sections were put in working elastic stain solution (20ml hematoxylin solution, 3ml ferric chloride solution, 8ml Weigert’s iodine solution and 5ml deionized water) for 10 minutes before rinsing in deionized water. Next, sections were differentiated in working ferric chloride solution (3ml ferric chloride solution and 37ml deionized water), rinsed in tap water and checked microscopically. Then sections were rinsed in 96% ethanol to remove iodine, followed by deionized water. Van Gieson staining solution was applied for 2 minutes and lastly sections were rinsed in 96% ethanol before dehydration to n-butyl acetate and mounted with Entellan (Merck, Darmstadt, Germany).

Masson Trichrome Goldner (MTG) staining (Merck, Darmstadt, Germany) was performed according to manufacturer’s protocol with modifications in the incubation times. Briefly, Weigert’s iron hematoxylin staining solution (Weigert’s solution A and B, 1+1, Merck, Darmstadt, Germany) for staining of cell nuclei was applied for 8 minutes to the specimens followed by running tap water for 5 minutes. Before and after each of the following staining steps, sections were rinsed in 1% acetic acid for 30 seconds. Sections were first put in Azophloxine solution for 45 minutes prior to incubation in Tungstophosphoric acid orange G solution for 1 minute. Lastly, sections were stained with Light green SF solution for 2 minutes. After the last washing step with 1% acetic acid sections were dehydrated with a graded alcohol series from 70% ethanol to n-butyl acetate and mounted with Entellan subsequently.

For Picrosirius red staining, sections were first put in Weigert’s iron hematoxylin for 8 minutes prior to 5 minutes tap water to stain cell nuclei. Afterwards, sections were incubated in 0.1% Picrosirius red solution for 1 hour and then washed two times in 0.5% acetic acid followed by dehydration from 70% ethanol to n-butyl acetate and mounted with Entellan. Image analysis of Picrosirius red stained tissue was performed according to the protocol published by Vogel et al.[19]. Briefly, collagen (all types) exhibits a red fluorescence (Texas red channel) and elastic fibers as well as cellular material show green fluorescence (GFP-B channel).

Image acquisition was performed using a Nikon Eclipse Ti microscope (Nikon Instruments Austria). For all stainings, six pictures were taken in the medial layer at random spots (at a magnification of x40). Image analysis and quantifications were performed using NIS elements AR software.

### Fluorescence based stainings and auto-fluorescence based detections

For fluorescence-based stainings, antigen retrieval was performed in 1x tris(hydroxymethyl)aminomethane (Tris)/ethylenediaminetetraacetic acid (EDTA) after deparaffinization and rehydration of sections for 15 minutes at 98°C using a KOS microwave HistoSTATION. For staining the plasma membrane with wheat germ agglutinin (WGA) and 4′,6-diamidin-2-phenylindol (DAPI) as nuclei counterstain, sections were washed with 1x tris-buffered saline (TBS) two times for 5 minutes each. Sections were then incubated with CF555 WGA (Biotium, 5μg/ml in 1xTBS) for 15 minutes followed by three times of washing in 1xTBS/0.1% Tween20 (Biorad, California, USA). Of note, fluorescence-labelled WGA binds to sialic acid and N-acetylglucosamine in the plasma membrane [20]. Permeabilization was performed by applying 0.2% TritonX-100 in 1xTBS for 5 minutes. Afterwards, sections were washed two times in 1xTBS with 0.1% Tween20 for 5 minutes each. Blocking of unspecific binding sites was achieved by incubation of specimens in 10% normal goat serum with 1% bovine serum albumin (BSA) in 1xTBS. Subsequently, sections were washed in 1xTBS two times for 5 minutes prior to staining of nuclei with DAPI (Life technologies, 1μg/ml in 1xTBS) for 20 minutes. Finally, sections were washed three times for 5 minutes in 1xTBS and mounted using ProLong Gold antifade (Life technologies). Sections were stored at -20°C until image acquisition using a Nikon Eclipse Ti microscope. Six pictures were taken at random spots in the medial layer (at magnification x40). WGA as membrane positive signal (red channel) was calculated as area/0.1mm^2^ and cell nuclei (DAPI) were counted and calculated in native tissue per 0.1mm^2^ using NIS elements AR software. Of note, nuclei counted and depicted in decellularized tissue were no longer intact nuclei, but rather cell nuclei residues (residual DNA which is stained by DAPI).

For the quantification of the amount of elastin in the medial layer of the analysed tissues, an immunofluorescence staining was performed. Therefore, 5μm sections were washed two times with 1xTBS/0.1% Tween20 after antigen retrieval followed by permeabilization as described above. To block unspecific binding sites, sections were incubated with 5% BSA in 1xTBS for 30 minutes. Subsequently, primary anti-elastin antibody (mouse monoclonal, Santa Cruz, sc-58756, 2μg/ml) was incubated overnight at 4°C. After washing sections three times with 1xTBS/0.1% Tween20 for 5 minutes, secondary antibody incubation was performed using Alexa Fluor 546 goat-anti-mouse antibody (Molecular probes, A11030, 2.5μg/ml) for one hour at room temperature. Sections were then washed again three times with 1xTBS/0.1% Tween20. For the staining of nuclei, Hoechst 34580 (MedChemExpress, HY-15560B, 1μg/ml) was applied for 30 minutes at room temperature. After three final washing steps with 1xTBS, sections were mounted using ProLong Gold antifade. Image acquisition was performed using a Nikon Eclipse Ti confocal microscope supplemented with NIS elements AR software. Eight pictures were taken at random spots within the medial layer (magnification x60). Using NIS elements AR software, elastin expression was measured and quantified as area/1000μm^2^. Within the AAD type A group, the image acquisition for the evaluation of elastin (and elastic fibers as well as collagen) was performed only outside the dissection lamella within the medial layer.

To quantify the amount of elastic fibers, the auto-fluorescence properties of thick elastic fiber sheets were used and the area calculated. Therefore we established a detection- and quantification method based on the publications by Lee et al.[21] and Wong et al.[22], after an adoption as well as based on our experiences (auto-fluorescence based detection was performed in relation to results of the Elastika van Gieson staining to avoid misinterpretation). For this, sections were washed two times in 1xTBS/0.1% Tween20 for 5 minutes after antigen retrieval as described above. Specimens were then permeabilized using 0.2% TritonX-100 in 1xTBS for 5 minutes followed by two times of washing in 1xTBS/0.1% Tween20 for 5 minutes each. To block unspecific binding sites, specimens were incubated in 10% normal goat serum with 1% BSA in 1xTBS for 30 minutes. Lastly, sections were washed three times in 1xTBS for 5 minutes each and mounted using ProLong Gold antifade. Sections were stored at -20°C until image acquisition and analysis using Nikon Eclipse Ti microscope supplemented with NIS elements AR software. Six pictures of the media (at magnification x40) were taken in the green fluorescence channel at random spots and elastic fiber sheets (intense green) as well as other ECM components (less intense green) quantity were measured as area/1000μm^2^. The same exposure time was set for all images.

### Scanning electron microscopy

Samples were fixed in 2.5% glutaraldehyde EM grade (Agar Scientific, Stansted, Essex, UK) and stored at 4°C until further investigation. For dehydration, samples were first washed in PBS without CaCl_2_ and MgCl_2_ for 10 minutes followed by an increasing ethanol series (30%, 50%, 70%, 90%, three times absolute ethanol for 20 minutes each). Chemical dehydration was achieved by incubation of the samples with hexamethyldisilazane (HMDS) for 45 minutes and additional 10 minutes with fresh HMDS. Samples were then left to dry overnight before mounting on stubs. Conductive silver paint (Acheson DAG 1415, Plano) was painted around the samples for better conduction. Before analysis, samples were gold sputtered using Quorum Sputter Coater and pictures were taken using JEOL LSM 5400 and Zeiss EVO10 SEM.

### Statistical analysis

Statistical analyses were performed using SPSS 24.0 software and graphical presentation of box plots were performed using GraphPad Prism 6.04 software. First, all data were analysed for distribution (Gaussian distribution). Depending on the data distribution, parametric (paired t-test) or non-parametric (Wilcoxon test) statistical tests for dependent samples were performed to test for differences between native and decellularized tissue of the related groups. To test for differences in mean values between non-aneurysmal and aneurysmal/dissection tissue within the two groups (native and decellularized) a t-test for independent samples or a Mann-Whitney-U test was performed. A p-value of ≤0.05 was assumed as a significant difference. For further information regarding statistical tests performed and p-values calculated we refer to Table A in S1 File and Table B in S1 File in the Supporting information S1 File. Raw data can be found in the Supporting information S2 File.

## Results

### Successful decellularization of aneurysmal- and dissection aortic tissue and corresponding control specimens using sodium hydroxide

**Fig 1:**
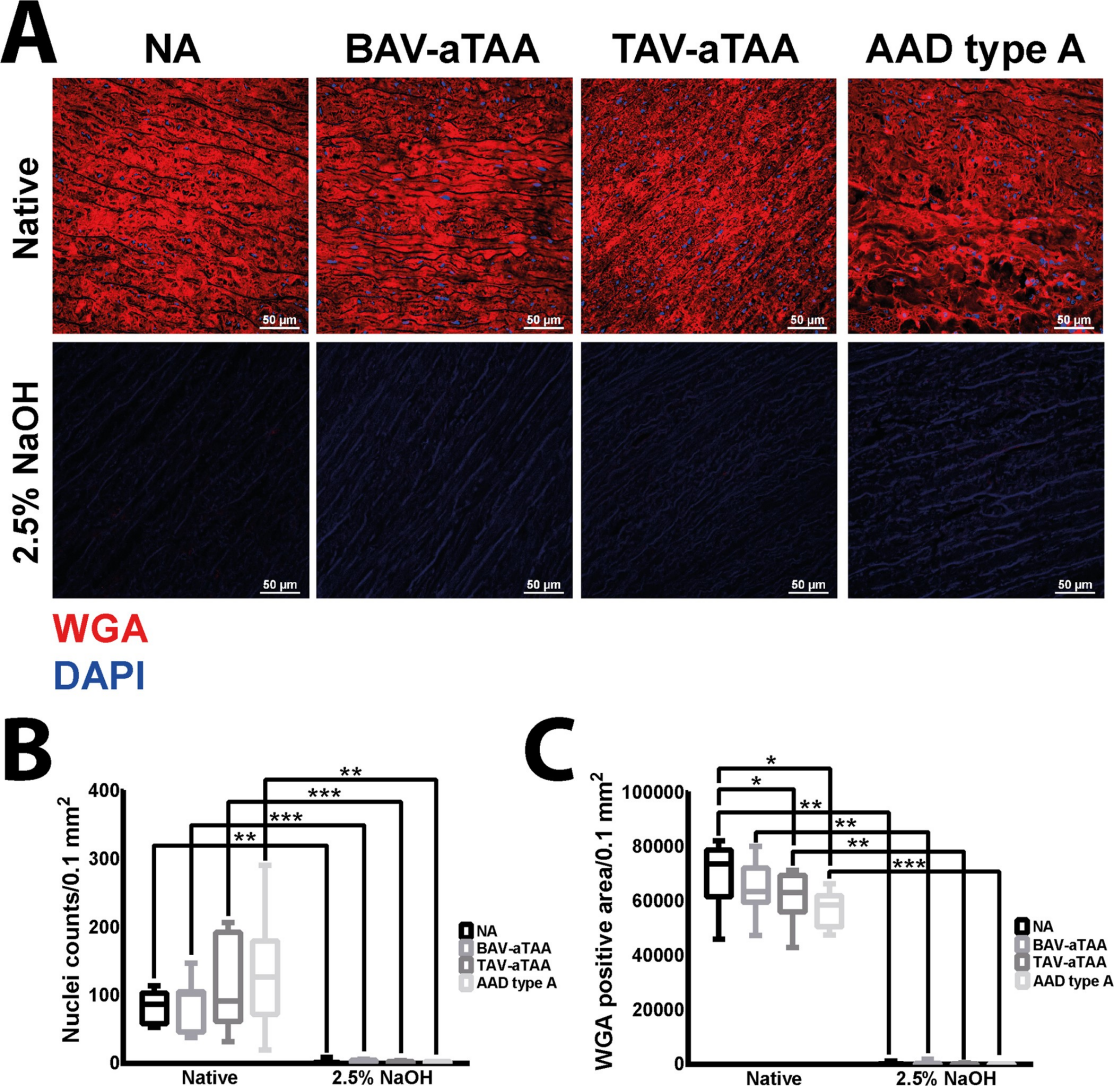
Determination of decellularization extent in NA as well as aneurysmal/dissection tissue of different origin. In (A) upper row, the native tissues of the corresponding groups are shown. In blue the cell nuclei are depicted and in red the plasma membrane is shown by WGA staining. In the row below, representative images of corresponding decellularized tissues are depicted. Scale bar represents 50μm. (B) Quantification of cell nuclei and (C) WGA positive staining are shown using box plots with mean and standard deviation. NA n = 9; BAV-aTAA n = 9; TAV-aTAA n = 9; AAD type A n = 8. * p<0.05, ** p<0.01, *** p<0.001.

In order to check for complete decellularization using 2.5% NaOH, a nuclear staining using DAPI and staining of plasma membrane with WGA (wheat germ agglutinin, CF555 conjugate) was performed. Fluorescence-based stainings depicted in Fig 1A (upper row) show that in the native tissue cell nuclei (blue) and plasma membrane (red) were present in all samples. However, after incubation with 2.5% NaOH for 48 hours neither cell nuclei nor plasma membrane were present in control and diseased tissue (Fig 1A, second row). Of note, cell nuclei counted in decellularized tissues were no longer intact and are to be seen as remnants. Quantification of the cell nuclei numbers revealed a significant reduction in decellularized tissue compared to native tissue in all analysed tissue samples (Fig 1B). Likewise, the red staining corresponding to the plasma membrane was quantified. Fig 1C shows that in all tissue samples, plasma membranes were successfully removed after 48 hours of incubation with the alkaline solution. Aside from decellularization-dependent reduction in WGA stained plasma membranes in decellularized tissue samples compared to native tissue, interestingly a disease related reduction in the amount of sialic acid and N-acetylglucosamine (which is determined by quantifying the WGA signal since WGA binds to sialic acid and N-acetylglucosamine) was observable in TAV-aTAA and AAD type A patients compared to the NA tissue within the native non-decellularized tissues (Fig 1C).

### Disease-related changes in the content of ECM components in native tissue and decellularization dependent changes in ECM quality

**Fig 2:**
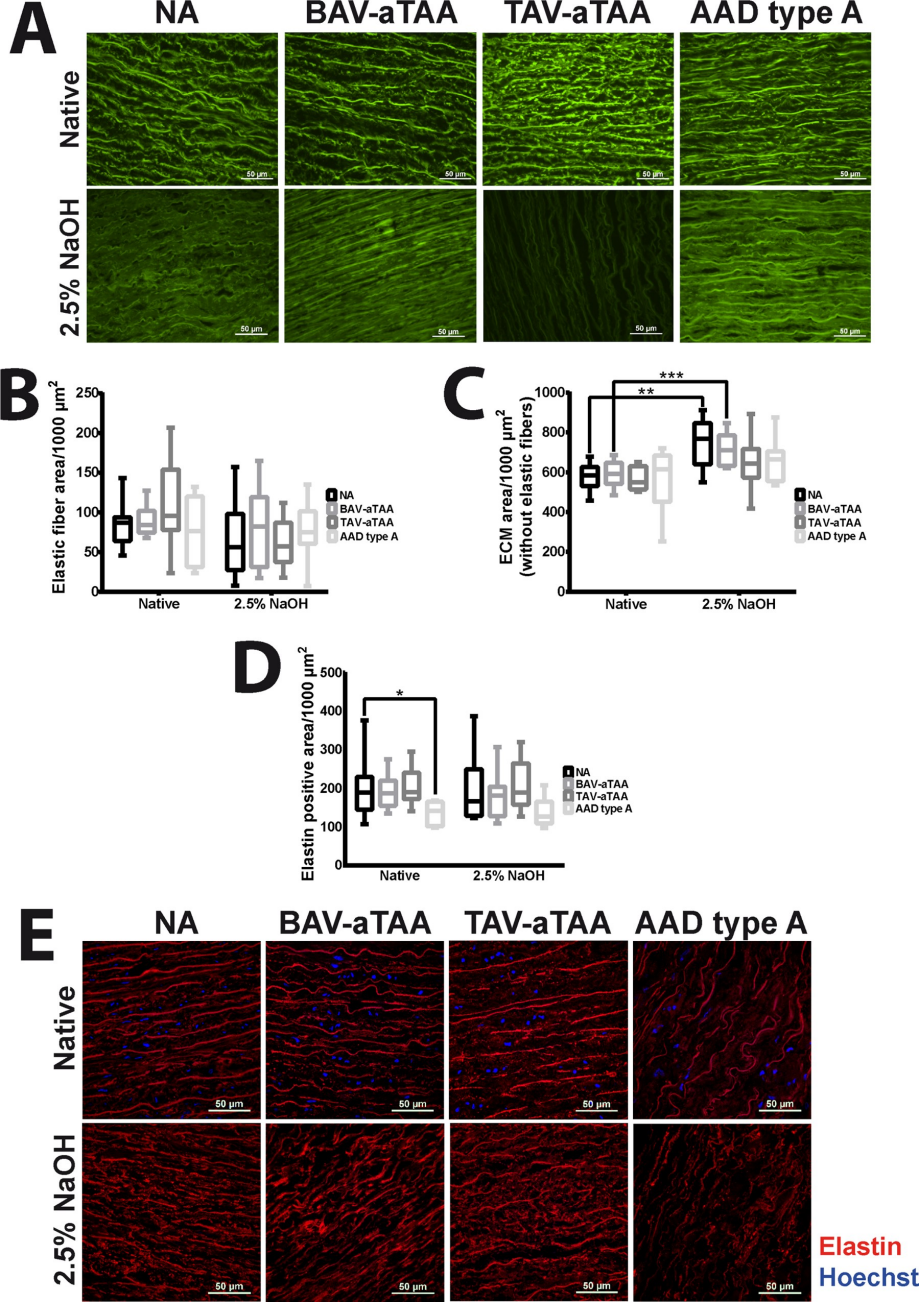
Quantification of ECM components of NA and aneurysmal/dissection tissues. (A) Representative images of the auto-fluorescence properties of elastic fibers and other ECM components to quantify the ECM of native and decellularized tissue are shown (elastic fibers with stronger auto-fluorescence in GFP-B channel compared to residual ECM). Scale bar represents 50μm. In (B) and (C) the quantification of elastic fiber area as well as ECM area without elastic fibers are shown in box plots with mean and standard deviation. Box plots in (D) show the quantification of elastin expression in native as well as decellularized NA and aneurysmal/dissection tissue. (E) shows representative images of antibody specific elastin staining (shown in red) in native and decellularized NA and aneurysmal/dissection tissue. Cell nuclei depicted in blue are stained with Hoechst. NA n = 9; BAV-aTAA n = 9 and for elastin n = 8; TAV-aTAA n = 9; AAD type A n = 8. * p<0.05, ** p<0.01, *** p<0.001.

To quantify the content of elastic fiber sheets and other ECM components, the different auto-fluorescence properties of elastic fibers and other ECM components were used for investigation and quantification. Elastic fibers exhibit a more intense fluorescence in the green fluorescence channel (GFP-B) than other ECM parts. Both, the representative images of auto-fluorescence properties of elastic fibers as well as other ECM components (Fig 2A), and the quantifications of the corresponding signals shown in Fig 2B and 2C revealed that the amount of elastic fibers was not significantly changed, neither in native aneurysmal/dissected tissue versus native NA tissue nor in the decellularized tissue versus the native tissue. However, the comparison of representative images of native and decellularized TAV-associated aneurysmal tissue gives a hint for serious qualitative impairment of elastic fibers shown by the strongly decreased auto-fluorescence signal in decellularized tissue as compared to native tissue. It seems that the decellularization method reduces the auto-fluorescence properties of mainly elastic fibers, which is perhaps based on a disease-related prior impairment of elastic fibers that is only visible after decellularization in aortic tissue of TAV patients. The increase of the ECM area in the decellularized samples compared to the native samples (with a significant difference in NA and BAV-aTAA tissues) cannot be interpreted as an increase of the content, but is probably due to a better visibility of the ECM (and thus increased signal) after the decellularization process and the associated removal of the cellular components (Fig 2C). Aside from auto-fluorescence based detections, antibody-mediated detection of elastin expression was performed. As shown in Fig 2D, quantification of elastin positive area revealed disease-related and significant reduction in elastin expression in native AAD type A tissue compared to native NA tissue. Comparison of elastin expression profiles between native and decellularized tissue suggests no influence on elastin protein quality, as contact with the alkaline solution does not reduce the elastin amount in diseased groups. Representative images of elastin stained (shown in red) non-aneurysmal, aneurysmal and dissection tissues are displayed in Fig 2E. Cell nuclei are stained in blue and are only present in the native tissues.

### Histological stainings substantiate decellularization efficacy and demonstrate aortic structure before and after decellularization

**Fig 3:**
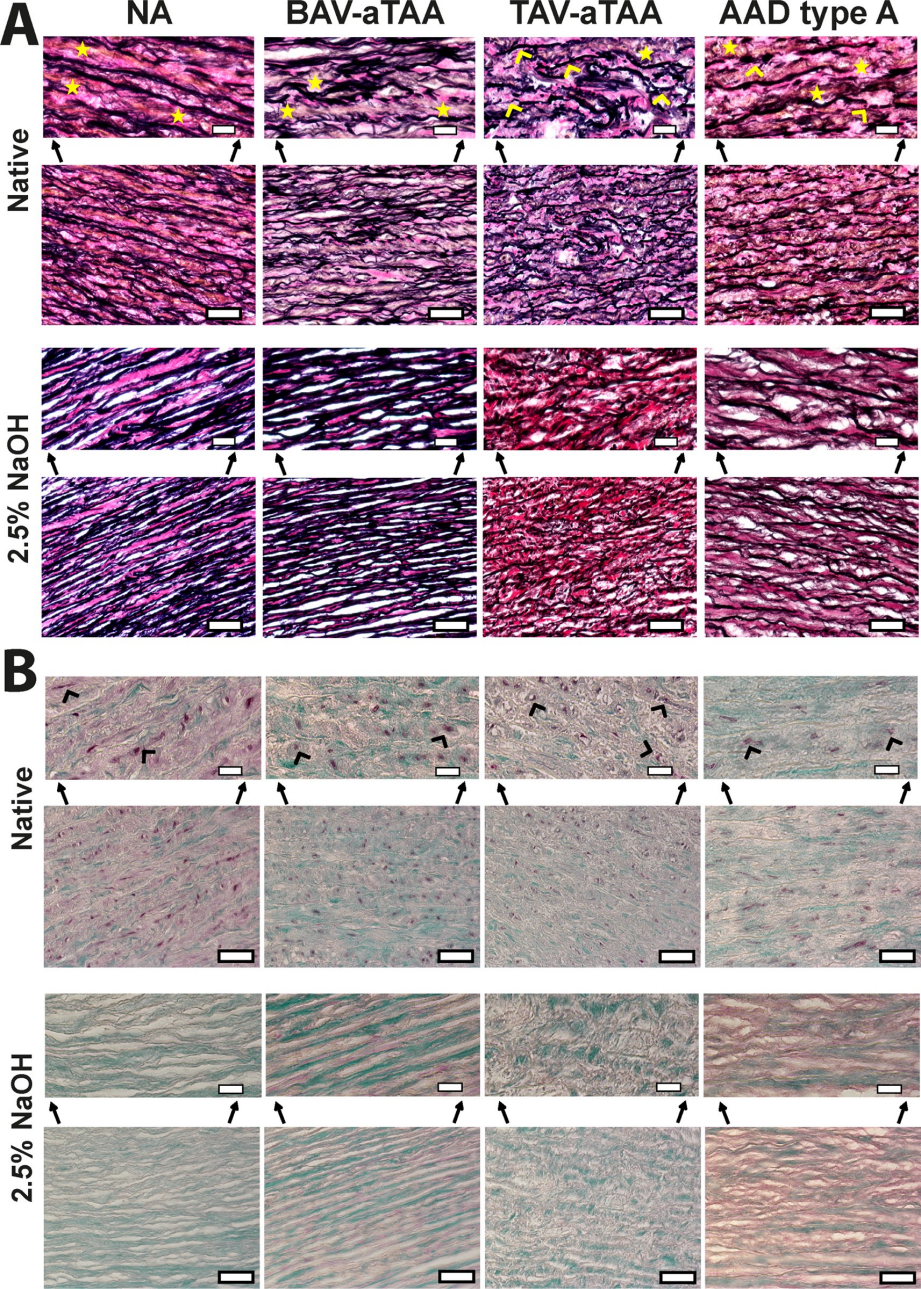
Elastika van Gieson and Masson Trichrome Goldner stained aortic NA and aneurysmal/dissection tissue. In (A) representative images of Elastika van Gieson stained NA and aneurysmal aortic tissue, either native (upper row) or decellularized (with 2.5% NaOH; lower row), are shown as well as magnified areas of the representative images. Yellow asterisks represent muscle cells and yellow arrowheads elastic fibers. In (B) representative images as well as magnified areas of Masson Trichrome Goldner stained aortic tissue are shown. The upper row shows native tissue of NA specimen, aneurysmal tissue of BAV- or TAV-origin as well as dissection specimen. Black arrowheads indicate cellular material. In the row below the corresponding decellularized tissues are depicted. Scale bar represents 50μm and in the magnified areas 20μm.

To further prove the decellularization efficacy and to gather more information regarding structural differences either induced by decellularization or present as disease-related changes, histological stainings were performed. In Fig 3A representative images and also magnified areas (shown above the images) of Elastika van Gieson stained aortic tissue, native (upper row) or decellularized (lower row) are shown. As already auto-fluorescence based evaluations showed, staining of elastic fibers (black) and collagen (reddish to pink) prove complete decellularization as well as disease-related structural differences (not due to decellularization method) mainly present in TAV-aTAAs (seen by thinner and disrupted elastic fibers, marked with yellow arrowheads). Likewise, also medial aortic tissue of AADs type A exhibits disease-related irregular elastic fibers (Fig 3A, marked with yellow arrowheads). Muscle cells stained in light yellow were present in native tissues (marked with yellow asterisks for better visibility), although it seems that the amount of this cellular wall part is reduced in native TAV-aTAA tissue compared to the other three groups. Regarding collagen, stained in reddish-pink, pictures shown in Fig 3A raised the concerns about an effect of the decellularization protocol on the amount of this structural protein, especially in the diseased groups.

Secondly, MTG stainings were performed to characterize cellular and extracellular parts of the aortic tissue in more detail (Fig 3B). Cell nuclei stained in brown were found only in native tissue and are absent after detergent treatment for all samples, showing complete decellularization. MTG staining shows viable tissue/cells in reddish colour. Images and magnified areas in Fig 3B show that the amount of cellular material is reduced in decellularized tissue (lower row) as compared to native tissue (upper row; shown by reduced reddish staining within the decellularized tissue compared to the native tissue, marked with black arrowheads within the native tissue images). In line with the Elastika van Gieson stained tissues, also this histological staining confirms the complete decellularization of the tissue. Nevertheless, this staining gives the impression that the amount of viable tissue parts is higher in NA tissue compared to aneurysmal/dissection tissue (Fig 3B, first row). Regarding the amount of ECM components, stained in blue/green, it seems that decellularization had no effect on the amount (second row). However, this staining suggests that structural impairment in AAD type A samples is only visible after decellularization (Fig 3B, second row).

### Quantitative measurements revealed a decellularization-related reduction in collagen content only in BAV-aTAAs and TAV-aTAAs

**Fig 4:**
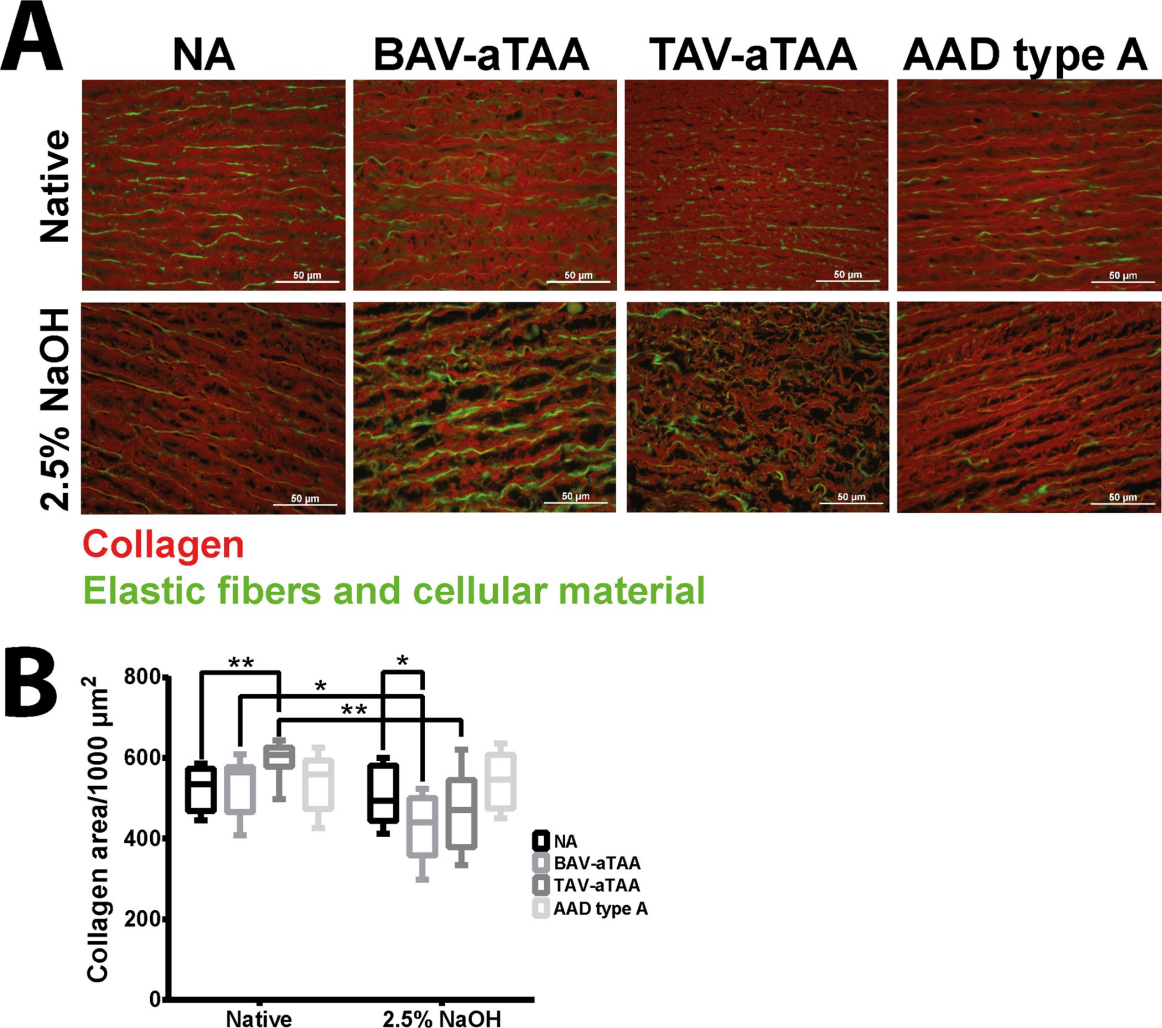
Determination of aortic collagen content before and after decellularization. In (A) representative images of Picrosirius red stained aortic NA and aneurysmal/dissection tissue after image acquisition using a fluorescence microscope are depicted (collagen in red, elastic fibers and cellular material in green). The upper row shows the native tissue und the lower the tissue after decellularization. Scale bar represents 50μm. (B) shows quantification of medial collagen content in native and decellularized NA tissue as well as aneurysmal/dissection tissue. NA n = 9; BAV-aTAA n = 9; TAV-TAA n = 9; AAD type A n = 8. * p<0.05, ** p<0.01.

To quantify the amount of collagen, we used the method described by Vogel et al.[19]. As depicted in representative images in Fig 4A, collagen captured in the Texas red fluorescence channel, can be seen in red in native NA tissues, BAV-tissue, TAV-tissue and AAD type A-tissue as well as after decellularization using 2.5% NaOH. Elastic fibers and cellular material are shown in green. Contrary to the assumption about the unchanged amount of ECM made after the examination of the Elastika van Gieson staining, significantly reduced amount of collagen was found after decellularization of BAV-aTAAs and TAV-aTAAs compared to native tissue (quantification of red signal in Fig 4B) using this collagen quantification method. In this case, collagen already damaged by the disease is most likely attacked by the alkaline decellularization solution. No difference was observed for NA tissues and AADs type A between native tissue and after decellularization (Fig 4B). Interestingly, in native tissue an increased collagen amount was seen in TAV-aTAA compared to NA tissue. After decellularization significant less collagen is present in BAV-aTAA tissue compared to NA tissue (Fig 4B).

### Structural changes in aneurysmal/dissection tissue evaluated by scanning electron microscopy

**Fig 5:**
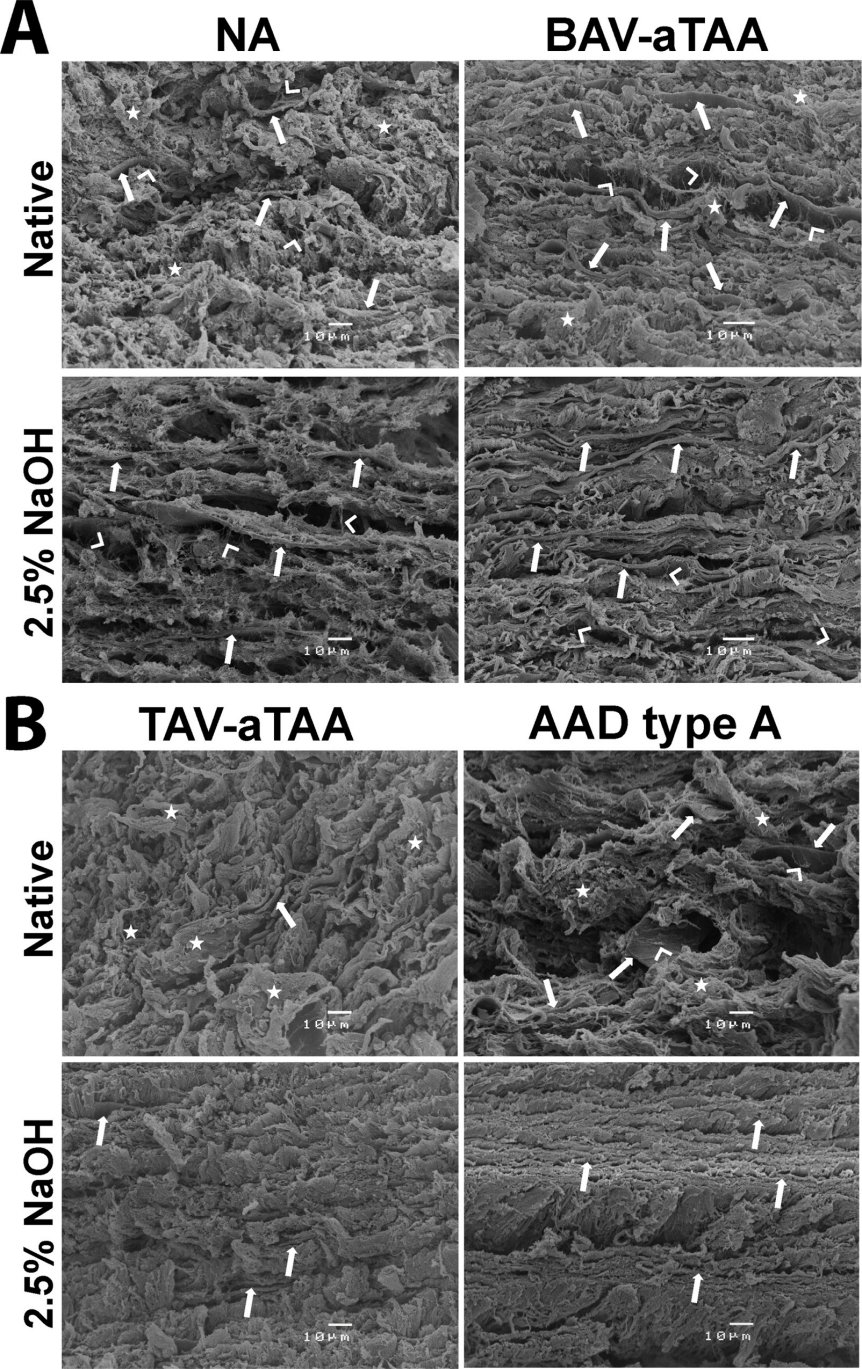
SEM based evaluation of elastic fiber sheets and other ECM components of NA and aneurysmal/dissection tissues. In (A) representative SEM pictures of native (upper row) and decellularized tissue (lower row) of controls (NA) and BAV-aTAAs are shown. In (B) SEM pictures of native and decellularized TAV-aTAAs tissue as well as tissue from a patient with an aortic dissection (AAD type A) are shown. White arrows are indicating elastic fibers, white asterisks are indicating cellular material, and white arrowheads are indicating interlaminar elastin fibers.

To evaluate the 3D structure of the medial layer of NA and aneurysmal tissues, native and decellularized samples were analysed by SEM (Fig 5). Since the identification of individual ECM components in electron microscopy (without antibody-based immunogold labelling) is not possible, we mainly focus here on the clearly recognizable and identifiable elastic fiber sheets. In NA tissue samples (Fig 5A, upper row) elastic fibers (arrows) were partly visible in native tissue and surrounded by ECM fibers and SMCs (asterisks). The structure of elastic fibers appearing as parallel arranged sheets was clearer visible after decellularization using 2.5% NaOH. Interlaminar elastin fibers (marked with white arrowheads) present between the elastic fiber sheets, as already described by Ushiki et al.[23] were clearly identifiable in native as well as decellularized NA and BAV-aTAA tissue. Regarding TAV-aTAA tissue, these characteristic interlaminar elastin fibers are not identifiable in neither native nor decellularized tissue (Fig 5B, left side). In contrast, these interlaminar elastin fibers can be seen in the native AAD type A tissue although the structure, especially of the media, is generally disordered and impaired. In decellularized tissue, these interlaminar elastin fibers are not visible at all because the structure appears fused in AAD type A tissue compared to NA tissue (Fig 5B, right side). Elastic fiber sheets were observed in native tissue of BAV-aTAAs with similar structure as in NA tissues and cellular material was found in between elastic lamellae (Fig 5A, upper row, arrows). Even after NaOH treatment, elastic fibers were clearly visible as parallel sheets with no obvious sign of degeneration (Fig 5A, second row). ECM components other than elastic fiber sheets showed the same regular distribution as in the NA tissues. In contrast, in the medial layer of TAV-aTAAs elastic fiber organization appeared more irregular and degraded compared to NA tissue (Fig 5B, first row). Elastic fibers were only partly visible and not as compact disks as in NA tissue. Cell removal from TAV-aTAA tissue improves visibility on elastic fiber sheets, but nonetheless compact elastic fiber disks are disorganized when present but also only sporadically present (Fig 5B, lower row). Moreover, ECM components other than elastic fibers seem to be more disorganized in TAV-aTAA tissue. For AADs type A, within native tissue elastic fiber sheets are present however also more disorganized compared to the NA tissue (Fig 5B, upper row). Interestingly, after decellularization the SEM based detection of ECM quality in AAD type A tissue changed completely. Cell removal leads to complete elastic fiber disk collapse and a kind of fusion of elastic fibers with other ECM components (Fig 5B, second row).

**Fig 6:**
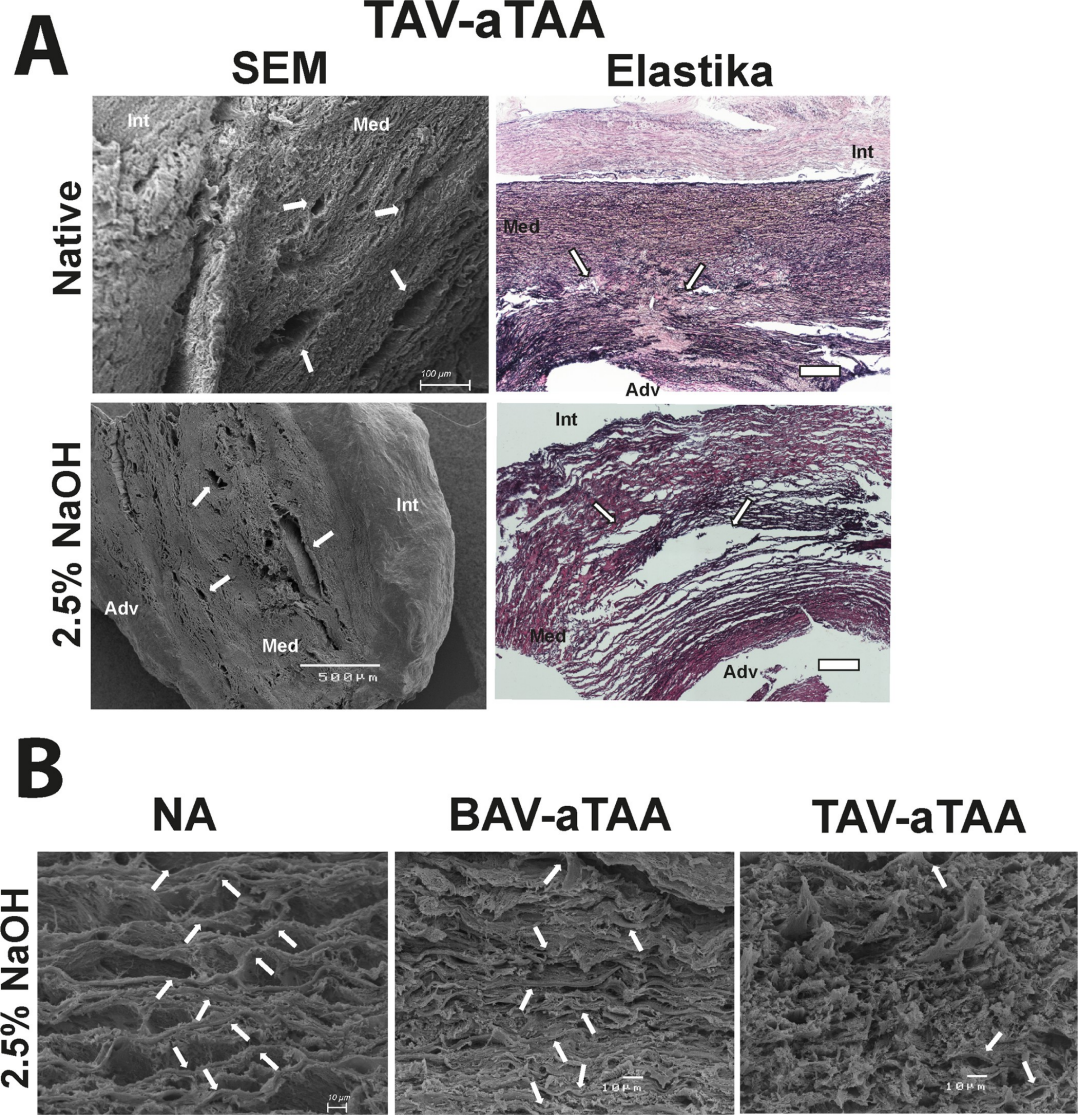
Comparison of SEM pictures with histological stainings and higher magnification pictures to show elastic fiber sheets after decellularization. In (A) the focal medial degeneration (white arrows) of TAV-aTAAs is depicted by SEM images as well as in Elastika van Gieson stained tissue. Scale bar in the Elastika van Gieson staining represents 250μm. (B) Representative SEM images of NA and aneurysmal tissue after decellularization (white arrows are indicating elastic fiber disks). Int = intima; Med = media; Adv = adventitia.

Furthermore, the TAV-aTAA typical focal medial degeneration of elastic fibers observable in the Elastika van Gieson staining is clearly detectable also in SEM pictures of native and decellularized tissue (Fig 6A, white arrows). SEM based observations also showed that the number of elastic fiber sheets is reduced in TAV-aTAAs compared to NA and BAV-associated tissue (Fig 6B, white arrows). Moreover, also the higher magnification pictures in Fig 6B are demonstrating that the ECM structure and its components are completely disorganized in TAV-forms compared to BAV ones and NA tissue, even in places where no strong media degeneration is present.

**Fig 7:**
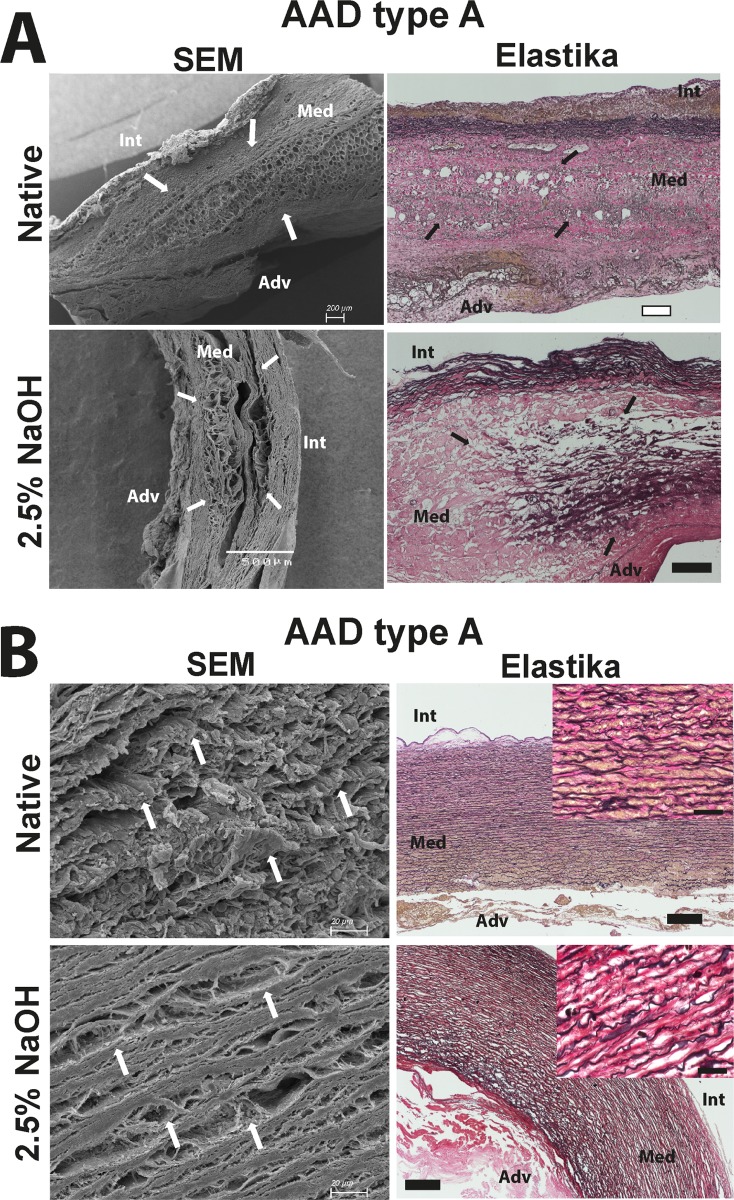
Representative images indicating the dissection lamellae and the ECM structure of aortic dissection specimens aside from the dissection lamellae. In (A) overview pictures of the dissection lamellae (white/black arrows) before and after decellularization are shown using SEM-based image acquisition and the corresponding tissue specimen after Elastika van Gieson staining. (B) Representative SEM images of the ECM structure in native and decellularized dissection tissue aside from the dissection lamellae and the corresponding pictures of Elastika van Gieson staining. White scale bar represents 200μm and black scale bar 250μm. Scale bar in the small pictures (magnification x40) represents 50μm. Int = intima; Med = media; Adv = adventitia.

To show structural differences within the AAD type A group, we depicted two samples in Fig 7. Fig 7A shows electron microscopic pictures as well as Elastika van Gieson stained slides of native and decellularized AAD type A tissue near the dissection lamellae (Fig 7A, white/black arrows). At side of dissecting media, a complete destroyed structure is detectable in histological as well as in SEM pictures (for both, native and decellularized tissue). In contrast, aortic tissue from AAD patients apart from dissection lamellae has a histological structure that is normal throughout and comparable to that of controls, even after decellularization of the tissue (Fig 7B, Elastika van Gieson staining). However, electron microscopic pictures (Fig 7B, SEM) revealed that thin individual ECM components are not clearly detectable and seemed to be melted together and even together with the elastic fiber sheets (as compared to NA and also aneurysmal tissue). This fact becomes even more striking when the tissue is decellularized. The histological staining does not differ from that of control tissue, but the fusion of the thin fibers displayed in the electron microscope is very strong (Fig 7B, decellularized tissue, white arrows point to melded thin fibers).

## Discussion

The organization of the aortic ECM is very crucial since it is needed for maintaining tensile strength as well as stiffness of the aorta.[15,16,24] Any alterations in the composition of the ECM including collagen and elastin have tremendous effects on the normal function of the ECM which might be involved in the formation of aneurysms or dissections.[25–28] Therefore the present study aimed at analysing the ECM structure/amount/quality of control and aneurysmal/dissection tissue in its native form as well as after decellularization. Using decellularization of aneurysmal and dissection tissue by an alkaline solution (NaOH), subsequent histological and detailed SEM examinations, we describe so far unreported and potential disease-related changes in elastic fiber and collagen quantity/quality apparent in native tissue or which become only evident after contact with alkaline solution. Of note, comparison of native with decellularized and non-aneurysmal with aneurysmal/dissected tissue is necessary to prevent misinterpretation of the data.

Development of aneurysms and dissections are an increasing health problem affecting more and more younger people.[29] However, apart from known mutations, the underlying causes are still unknown in the vast majority of cases.[4,30] Especially the ECM with its individual components is highly important for proper aortic wall function.[26] A disruption of this functional balance, as it is known especially for TAV-associated aneurysms [5,31], is perhaps involved in disease development and certainly in the progression of disease. A detailed characterization of pathological changes affecting the ECM potentially helps to unravel the underlying causative disease-initiating factors. Particularly with regard to rarer aneurysm forms such as BAV-aTAAs and the development of dissections, not much is known about the structural changes.[13,32]

In general, examination of ECM is easier to perform without the presence of cells. Therefore, we searched in preliminary tests for a decellularization protocol to be highly effective in decellularization with simultaneous minimal effect on the ECM and its components. As previously published by Borges et al.[17] we also used NaOH for decellularization, however in a substantial lower concentration of 2.5% (0.6N versus 2N NaOH solution). Since we intended only to achieve cell free aneurysmal/dissection and control tissue we reduced the concentration to a minimum compared to Borges et al.[17] who intended to remove elastin and analyse only collagen. As expected, we successfully removed the cellular parts of aortic non-aneurysmal and aneurysmal/dissection tissues, since neither cell nuclei nor plasma membrane components were present. In summary, the decellularization method used has an effect on ECM. But a comparison with the corresponding native tissue as well as native and decellularized NA tissue (and the changes that do not occur there) shows that the changes caused by decellularization can be an indication of the disease-related limited quality of the ECM. Thus, we see the treatment of the tissue with an alkaline solution at a low concentration not only as pure decellularization, but as a possibility to evaluate the quality of ECM components. The increase in the amount of ECM in decellularized NA and BAV-aTAA tissues compared to corresponding native tissues can be explained by cell removal which facilitates the detection of the ECM underneath the cells and is therefore responsible for the increase (present in nearly all groups, statistically significant in NA and BAV-aTAA groups). The overall absent difference in elastic fiber content between NA tissue and aneurysmal tissue can be explained by the mainly focal and not generally dispersed loss of elastic fibers in aneurysmal tissue.[31] Of note, results of auto-fluorescence based elastic fiber detection and quantification of elastin expression cannot be directly compared with each other as elastin is not only part of elastic fibers, but elastin is also expressed as interlaminar elastin fibers (connecting two adjacent elastic fiber sheets) and elastin struts as well as elastin microfibrils.[16]

BAV-associated aTAAs are known to grow faster and are present at a younger age.[33,34] Comparison of native tissue samples from BAV patients with corresponding NA tissue revealed no pathological change, neither in elastic fiber content, elastin expression, in the amount of other ECM components together, nor in separate collagen content. Moreover, electron microscopic evaluations revealed no evident difference in the quality of ECM components compared to NA tissue. However, after decellularization the BAV-associated tissue showed an impairment of medial collagen content, expressed as a significant reduction in the amount compared to native as well as decellularized NA tissue. As this reduction of collagen content is not present in decellularized NA tissue we can only speculate about a disease-related impairment of aortic medial collagen quality which only becomes evident after contact with the alkaline solution in the BAV-aTAA group. However, it seems that this effect is restricted to collagen. Relying on previous publications from Wågsäter D et al.[35], Bode M.K. et al.[36], and Lindeman J.H.N. et al.[37], which showed an impairment of collagen microarchitecture in human abdominal as well as BAV-associated ascending aortic aneurysm, we hypothesize that disease-associated defective collagen synthesis/fibrillogenesis/microarchitecture impairs the integrity of collagen and thus makes it accessible for degradation by alkaline solutions resulting in the reduced amount of this ECM protein. Furthermore, based on the fact that collagen dissolves in alkaline solutions [38], it is conceivable that collagen in aneurysmal tissue can be dissolved more easily by alkaline solutions. In summary, this is a novel result, as structural changes within the BAV aneurysm group are hardly seen until now.[39] These results can be linked to the results of previous studies.[35–37]

Interestingly, although known as a completely different disease with the same outcome, the emergence of an enlarged aorta [4], the group of TAV-aTAAs likewise shows a reduction in collagen content after decellularization. Therefore, we hypothesize also about a disease related impairment of collagen fiber quality which becomes evident after contact with the alkaline solution. Of note, the amount of collagen in native TAV-aTAA tissue is higher as compared to NA tissue. An increased expression of collagen I and II was already described by Meng et al.[40] for aTAAs, although there are results to the contrary [8]. But contact with the alkaline solution even reduces the amount nearly to the level of decellularized NA tissue. However, it should be added that in contrast to the BAV-aTAAs in the TAV-associated forms, a strong impairment of the ECM in the electron microscope can still be detected, although this is not so visible from the histological analyses. As already mentioned, the overall amount of elastic fibers is unchanged in native TAV-aTAA patients as compared to the native NA tissue. Likewise no change is observable in decellularized TAV tissue compared to native tissue. It seems that elastic fiber sheet loss is more focal than generally distributed, as already described by Borges et al.[31]. This is particularly important for the analysis of the TAV-aTAA group because it has been proven that this group has a strong disease-related impairment of the elastic fibers.[5,7,31] Nevertheless, the obvious loss in auto-fluorescence properties of elastic fibers in this aneurysm group after contact with alkaline solution (which is not present to this extent in the other aneurysmal group, the NA group and dissected tissue) argues probably for a disease-related impairment of elastic fiber quality which became apparent after decellularization. As Borges et al.[17] were able to remove elastic fibers with alkaline solution, we hypothesize that our lower concentrated alkaline solution affects only prior impaired elastic fibers in diseased tissue. Remarkable, elastin expression within the aortic media of TAV patients is unchanged compared to NA tissue and not affected by decellularization. So the reduced auto-fluorescence of elastic fibers and the proposed disease-related impaired quality of these sheets does not appear to be due to an effect on the elastin content or its stability. Again, these are important findings which make clear that some differences are more hidden and that several methods of verification should always be used.

As already stated above, descriptions about the signalling pathways involved in aortic dissection development or at least the analysis of structural changes in the aortic media of dissection patients are unfortunately very rare.[41] Data regarding the structural changes and ECM composition of dissecting aortic tissue are still much rarer available.[41,42] However, as it becomes increasingly clear that dissections are not a consequence of aneurysms but an own disease [13], further studies are needed to prove this. However, data are also urgently needed explaining the development and presence of pathological changes since the number of affected patients of this disease is also increasing. Beyond, persons affected by an type A AAD are also getting younger.[13,43,44] Native AAD type A tissue also showed no overall pathological changes in regard to elastic fiber content, in the area of other ECM components, and separate collagen content as compared to NA tissue. However, elastin expression is pathologically reduced in tissues of AAD type A patients as compared to NA subjects. But this is not a new result, as a reduced/impaired expression and assembly of elastin is already described for AAD type A.[45–47] The unchanged amount of elastic fiber sheets and the simultaneous reduced expression of elastin in AAD type A patients can currently not be explained. Of note, the histological appearance of AAD type A medial aortic tissue very often differs greatly. Interestingly and in contrast to aneurysmal tissue of BAV- and TAV-origin, contact with NaOH in the course of decellularization procedure does not reduce the medial collagen content. However, SEM analyses also provided important new evidence and insights. The structure of ECM in the native as well as in the decellularized state was disordered in contrast to NA tissue. Additionally, a pathological change was observed both in the dissection lamella area and in the area beyond it. Nevertheless, the severity and quality of these changes varies. In the area of the dissection lamella a completely destroyed tissue is clearly visible, a heavy loss of elastic fibers and cellular material, which becomes particularly apparent after decellularization. In contrast, apart from the dissection lamella there are regular-looking elastic fiber plates. Nevertheless, a strong change of the fibrillary structure of the ECM in comparison to the NA tissue was already observed when looking at the native tissue. It becomes clear that the actual fiber-like structures have been lost and that these structures are now more like fused plates instead of fine fibers with a certain function. The presence of these fused fibers becomes even more apparent after decellularization of the dissection tissue. However, these changes are only visible by electron microscopic analysis and are not evident from histological images.

Potential disease-related changes in ECM as well as especially the loss in the quality of these components obvious after decellularization can be explained by a dysregulation in the matrix metalloproteinase/tissue inhibitor of metalloproteinases (MMP/TIMP) expression levels and patterns as already described.[48] Of note, differences in the manifestation of ECM changes between BAV- and TAV-associated aneurysm forms are potentially related to the characteristic and different MMP and TIMP expression patterns previously mentioned.[7,49–51] Likewise, changes in the ECM (e.g. the reduction in elastin expression) of dissection tissue described in this study can be connected to an already described increase in the MMP expression and changed MMP/TIMP expression ratios.[52–54] Likewise, the already proven increase in oxidative stress and the decreased expression of anti-oxidative enzymes (as for example superoxide dismutase) as well as the suggested link of this stress to dysregulated MMP function [55–58] represents an explanation for the impaired ECM quality shown in the present study.

## Conclusion

By decellularization of aneurysmal and non-aneurysmal tissue using an alkaline solution, differences in the quality of ECM components became obvious, which can partly be connected with already existing data. In terms of TAV-aTAAs, an impaired quality of elastic fibers became obvious, although the amount is not significantly reduced. Since this only occurs with TAV-aTAAs, this could indicate, apart from an already known focal loss of fibers, a previously unknown disease-related impairment of the elastic fiber quality, which of note is not due to a loss in elastin expression. Regarding collagen, a contact with alkaline solutions leads to a loss in the amount of this ECM protein, however only in aneurysmal tissue associated with a BAV or a TAV. As collagen dissolves after contact with alkaline solution and keeping in mind that we used a very low concentration, it is reasonable to assume that a previous disease-related damage to collagen causes the accelerated degradation in BAV- and TAV- associated aneurysmal tissue. The authors are completely aware that alkaline solutions are able to damage collagen and remove it from tissues.[38] However, the unchanged collagen content in the control tissue after decellularization (compared to native tissue) suggests that the differences in aneurysmal tissue after decellularization may indicate a pathological change in the quality of this protein and is no general effect of the alkaline solution treatment. Changes in the quality of ECM components within the ascending aorta of dissection patients were also detectable, however only in electron microscopic analyses.

However, it should be noted that observations of decellularized tissues in SEM and histological/auto-fluorescence stainings can only be interpreted with caution and in relation to the results from native tissue, since the decellularization may have an impact on ECM components. In summary, our results suggest that aside from the quantity, also the quality of ECM components within aneurysmal and dissection tissue should be analysed in more detail. That is what we learned from decellularization and electron microscopy of aneurysmal and dissection aortic tissue.

## Supporting information

S1 File Further information on statistical tests performed in this study. (DOCX)

S2 File These are the raw data of the publication. (XLSX)

## References

[1] WHO. Cardiovascular diseases (CVDs) [Internet]. 2018. Available: http://www.who.int/mediacentre/factsheets/fs317/en/#.V9U1f0lPSK0.mendeley

[2] CDC. Aortic Aneurysm [Internet]. Available: https://www.cdc.gov/dhdsp/data_statistics/fact_sheets/fs_aortic_aneurysm.htm

[3] Saeyeldin AA, Velasquez CA, Mahmood SUB, Brownstein AJ, Zafar MA, Ziganshin BA, et al Thoracic aortic aneurysm: unlocking the “silent killer” secrets. Gen Thorac Cardiovasc Surg. 2017; 10.1007/s11748-017-0874-x 29204794

[4] Isselbacher EM. Thoracic and Abdominal Aortic Aneurysms. Circulation. 2005;111: 816–828. 10.1161/01.CIR.0000154569.08857.7A 15710776

[5] Blunder S, Messner B, Aschacher T, Zeller I, Türkcan A, Wiedemann D, et al Characteristics of TAV- and BAV-associated thoracic aortic aneurysms—Smooth muscle cell biology, expression profiling, and histological analyses. Atherosclerosis. 2012;220: 355–361. 10.1016/j.atherosclerosis.2011.11.035 22178424

[6] Schmid F-X, Bielenberg K, Schneider A, Haussler A, Keyser A, Birnbaum D. Ascending aortic aneurysm associated with bicuspid and tricuspid aortic valve: involvement and clinical relevance of smooth muscle cell apoptosis and expression of cell death-initiating proteins. Eur J Cardiothorac Surg. Germany; 2003;23: 537–543. 12694773 10.1016/s1010-7940(02)00833-3

[7] Tang PCY, Coady MA, Lovoulos C, Dardik A, Aslan M, Elefteriades JA, et al Hyperplastic Cellular Remodeling of the Media in Ascending Thoracic Aortic Aneurysms. Circulation. 2005;112: 1098–1105. 10.1161/CIRCULATIONAHA.104.511717 16116068

[8] Borges LF, Jaldin RG, Dias RR, Stolf NAG, Michel J-B, Gutierrez PS. Collagen is reduced and disrupted in human aneurysms and dissections of ascending aorta. Hum Pathol. 2008;39: 437–443. 10.1016/j.humpath.2007.08.003 18261628

[9] Fedak PWM, De Sa MPL, Verma S, Nili N, Kazemian P, Butany J, et al Vascular matrix remodeling in patients with bicuspid aortic valve malformations: Implications for aortic dilatation. J Thorac Cardiovasc Surg. 2003;126: 797–806. 10.1016/S0022-5223(03)00398-2 14502156

[10] Cattell MA, Hasleton PS, Anderson JC. Increased elastin content and decreased elastin concentration may be predisposing factors in dissecting aneurysms of human thoracic aorta. Cardiovasc Res. England; 1993;27: 176–181. 8472268 10.1093/cvr/27.2.176

[11] Pasta S, Phillippi JA, Gleason TG, Vorp DA. Effect of aneurysm on the mechanical dissection properties of the human ascending thoracic aorta. J Thorac Cardiovasc Surg. Elsevier Inc.; 2012;143: 460–467. 10.1016/j.jtcvs.2011.07.058 21868041 PMC8084112

[12] Appelbaum A, Karp RB, Kirklin JW. Ascending vs Descending Aortic Dissections. Ann Surg. 1976;183: 296–300. 1259487 10.1097/00000658-197603000-00015 PMC1344241

[13] Golledge J, Eagle KA. Acute aortic dissection. Lancet. Elsevier; 2008;372: 55–66. 10.1016/S0140-6736(08)60994-0 18603160

[14] Erbel R, Alfonso F, Boileau C, Dirsch O, Eber B, Haverich A, et al Diagnosis and management of aortic dissection. Eur Heart J. England; 2001;22: 1642–1681. 10.1053/euhj.2001.2782 11511117

[15] Berillis P. The Role of Collagen in the Aorta’s Structure. Open Circ Vasc J. 2013;6: 1–8. 10.2174/1877382601306010001

[16] O’Connell MK, Murthy S, Phan S, Xu C, Buchanan J, Spilker R, et al The three-dimensional micro- and nanostructure of the aortic medial lamellar unit measured using 3D confocal and electron microscopy imaging. Matrix Biol. 2008/02/06 ed. 2008;27: 171–181. 10.1016/j.matbio.2007.10.008 18248974 PMC2679973

[17] Borges LF, Blini JPF, Dias RR, Gutierrez PS. Why Do Aortas Cleave or Dilate? Clues from an Electronic Scanning Microscopy Study in Human Ascending Aortas. J Vasc Res. 2014;51: 50–57. 10.1159/000356296 24335355

[18] Berryman MA, Porter WR, Rodewald RD, Hubbard AL. Effects of tannic acid on antigenicity and membrane contrast in ultrastructural immunocytochemistry. J Histochem Cytochem. 1992;40: 845–857. 10.1177/40.6.1350287 1350287

[19] Vogel B, Siebert H, Hofmann U. MethodsX Determination of collagen content within picrosirius red stained paraf fi n-embedded tissue sections using fl uorescence microscopy. MethodsX. Elsevier B.V.; 2015;2: 124–134. 10.1016/j.mex.2015.02.007 26150980 PMC4487704

[20] Murakami Y, Hasegawa Y, Nagano K, Yoshimura F. Characterization of wheat germ agglutinin lectin-reactive glycosylated OmpA-like proteins derived from Porphyromonas gingivalis. Infect Immun. 2014;82: 4563–4571. 10.1128/IAI.02069-14 25135681 PMC4249326

[21] Lee K-W, Stolz DB, Wang Y. Substantial expression of mature elastin in arterial constructs. Proc Natl Acad Sci. 2011;108: 2705–2710. 10.1073/pnas.1017834108 21282618 PMC3041142

[22] Wong LCY, Lowell LB. Developmental Remodeling of the Internal Elastic Lamina of Rabbit Arteries. Circ Res. American Heart Association; 1996;78: 799–805. 10.1161/01.RES.78.5.799 8620599

[23] Ushiki T. Collagen Fibers, Reticular Fibers and Elastic Fibers. A Comprehensive Understanding from a Morphological Viewpoint. Arch Histol Cytol. 2002;65: 109–126. 10.1679/aohc.65.109 12164335

[24] Avolio A, Jones D, Tafazzoli-shadpour M. Quantification of Alterations in Structure and Function of Elastin in the Arterial Media. Hypertension. 1998;32: 170–175. 10.1161/01.HYP.32.1.170 9674656

[25] Tsamis A, Krawiec JT, Vorp DA. Elastin and collagen fibre microstructure of the human aorta in ageing and disease: A review. J R Soc Interface. 2013;10 10.1098/rsif.2012.1004 23536538 PMC3645409

[26] Dingemans KP, Teeling P, Lagendijk JH, Becker AE. Extracellular matrix of the human aortic media: an ultrastructural histochemical and immunohistochemical study of the adult aortic media. Anat Rec. 2000;258: 1–14. 10.1002/(SICI)1097-0185(20000101)258:1<1::AID-AR1>3.0.CO;2-7 10603443

[27] Sariola H, Viljanen T, Luosto R. Histological pattern and changes in extracellular matrix in aortic dissections. J Clin Pathol. 1986;39: 1074–1081. 10.1136/jcp.39.10.1074 3537014 PMC500225

[28] Yamashiro Y, Yanagisawa H. Crossing bridges between extra- and intra-cellular events in thoracic aortic aneurysms. Crossing Bridg between extra- intra-cellular events Thorac aortic aneurysms. 2018;25: 99–110. 10.5551/jat PMC5827090 28943527

[29] Olsson C, Thelin S, Stahle E, Ekbom A, Granath F. Thoracic aortic aneurysm and dissection: increasing prevalence and improved outcomes reported in a nationwide population-based study of more than 14,000 cases from 1987 to 2002. Circulation. United States; 2006;114: 2611–2618. 10.1161/CIRCULATIONAHA.106.630400 17145990

[30] Vapnik JS, Kim JB, Isselbacher EM, Ghoshhajra BB, Cheng Y, Sundt 3rd TM, et al Characteristics and Outcomes of Ascending Versus Descending Thoracic Aortic Aneurysms. Am J Cardiol. 2016/03/27 ed. 2016;117: 1683–1690. 10.1016/j.amjcard.2016.02.048 27015890

[31] Borges LF, Gomez D, Quintana M, Touat Z, Jondeau G, Leclercq A, et al Fibrinolytic activity is associated with presence of cystic medial degeneration in aneurysms of the ascending aorta. Histopathology. 2010;57: 917–932. 10.1111/j.1365-2559.2010.03719.x 21166705

[32] Mordi I, Tzemos N. Bicuspid aortic valve disease: A comprehensive review. Cardiol Res Pract. 2012;1 10.1155/2012/196037 PMC3368178 22685681

[33] Grewal N, Gittenberger-de Groot AC, Poelmann RE, Klautz RJM, Lindeman JHN, Goumans M-J, et al Ascending aorta dilation in association with bicuspid aortic valve: A maturation defect of the aortic wall. J Thorac Cardiovasc Surg. 2014;148: 1583–1590. 10.1016/j.jtcvs.2014.01.027 24560417

[34] Tsamis A, Phillippi JA, Koch RG, Chan PG, Krawiec JT, D’Amore A, et al Extracellular matrix fiber microarchitecture is region-specific in bicuspid aortic valve-associated ascending aortopathy. J Thorac Cardiovasc Surg. 2016;151: 1718–1728.e5. 10.1016/j.jtcvs.2016.02.019 26979916 PMC4875874

[35] Wågsäter D, Paloschi V, Hanemaaijer R, Hultenby K, Bank RA, Franco-Cereceda A, et al Impaired collagen biosynthesis and cross-linking in aorta of patients with bicuspid aortic valve. J Am Heart Assoc. 2013;2: 1–11. 10.1161/JAHA.112.000034 23525417 PMC3603268

[36] Bode MK, Soini Y, Melkko J, Satta J, Risteli L, Risteli J. Increased amount of type III pN-collagen in human abdominal aortic aneurysms: Evidence for impaired type III collagen fibrillogenesis. J Vasc Surg. 2000;32: 1201–1207. 10.1067/mva.2000.109743 11107093

[37] Lindeman JHN, Ashcroft BA, Beenakker J-WM, van Es M, Koekkoek NBR, Prins FA, et al Distinct defects in collagen microarchitecture underlie vessel-wall failure in advanced abdominal aneurysms and aneurysms in Marfan syndrome. Proc Natl Acad Sci. 2010;107: 862–865. 10.1073/pnas.0910312107 20080766 PMC2818895

[38] Hey CD, Stainsby G. The enhanced solubility of collagen following alkaline treatment. Biochim Biophys Acta—Gen Subj. 1965;97: 364–366. 10.1016/0304-4165(65)90109-1 14292852

[39] Losenno KL, Goodman RL, Chu MWA. Bicuspid Aortic Valve Disease and Ascending Aortic Aneurysms: Gaps in Knowledge. Cardiol Res Pract. 2012;2012: 1–16. 10.1155/2012/145202 23198270 PMC3503270

[40] Meng Y, Tian C, Liu L, Wang L, Chang Q. Elevated expression of connective tissue growth factor, osteopontin and increased collagen content in human ascending thoracic aortic aneurysms. Vascular. 2014;22: 20–27. 10.1177/1708538112472282 23508392

[41] Nakashima Y. Pathogenesis of Aortic Dissection: Elastic Fiber Abnormalities and Aortic Medial Weakness. Ann Vasc Dis. The Editorial Committee of Annals of Vascular Diseases; 2010;3: 28–36. 10.3400/avd.AVDsasvp10002 23555385 PMC3595817

[42] Roberts WC, Vowels TJ, Kitchens BL, Ko JM, Filardo G, Henry AC, et al Aortic Medial Elastic Fiber Loss in Acute Ascending Aortic Dissection. Am J Cardiol. 2011;108: 1639–1644. 10.1016/j.amjcard.2011.09.005 22077975

[43] Howard DPJ, Sideso E, Handa A, Rothwell PM. Incidence, risk factors, outcome and projected future burden of acute aortic dissection. Ann Cardiothorac Surg. AME Publishing Company; 2014;3: 278–284. 10.3978/j.issn.2225-319X.2014.05.14 24967167 PMC4052413

[44] Melvinsdottir IH, Lund SH, Agnarsson BA, Sigvaldason K, Gudbjartsson T, Geirsson A. The incidence and mortality of acute thoracic aortic dissection: results from a whole nation study. Eur J Cardio-Thoracic Surg. 2016;50: 1111–1117. 10.1093/ejcts/ezw235 27334108

[45] Watanabe M, Sawai T. Alteration of Cross-Linking Amino Acids of Elastin in Human Aorta in Association with Dissecting Aneurysm: Analysis Using High Performance Liquid Chromatography. Tohoku J Exp Med. 1999;187: 291–303. 10.1620/tjem.187.291 10503601

[46] Cheuk BLY, Cheng SWK. Differential expression of elastin assembly genes in patients with Stanford Type A aortic dissection using microarray analysis. J Vasc Surg. 2011;53: 1071–1078.e2. 10.1016/j.jvs.2010.11.035 21276682

[47] Wang X, LeMaire SA, Chen L, Carter SA, Shen YH, Gan Y, et al Decreased expression of fibulin-5 correlates with reduced elastin in thoracic aortic dissection. Surgery. 2005;138: 352–359. 10.1016/j.surg.2005.06.006 16153447

[48] Tsarouhas K, Soufla G, Apostolakis S, Zaravinos A, Panagiotou M, Khoury M, et al Transcriptional regulation of TIMPs in ascending aorta aneurysms. Thromb Res. 2010;126: 399–405. 10.1016/j.thromres.2010.08.015 20863553

[49] LeMaire SA, Wang X, Wilks JA, Carter SA, Wen S, Won T, et al Matrix metalloproteinases in ascending aortic aneurysms: Bicuspid versus trileaflet aortic valves1. J Surg Res. 2005;123: 40–48. 10.1016/j.jss.2004.06.007 15652949

[50] Ikonomidis JS, Jones JA, Barbour JR, Stroud RE, Clark LL, Kaplan BS, et al Expression of matrix metalloproteinases and endogenous inhibitors within ascending aortic aneurysms of patients with bicuspid or tricuspid aortic valves. J Thorac Cardiovasc Surg. 2007;133: 1028–1036. 10.1016/j.jtcvs.2006.10.083 17382648

[51] Mohamed SA, Noack F, Schoellermann K, Karluss A, Radtke A, Schult-Badusche D, et al Elevation of Matrix Metalloproteinases in Different Areas of Ascending Aortic Aneurysms in Patients with Bicuspid and Tricuspid Aortic Valves. Sci World J. 2012;2012: 1–7. 10.1100/2012/806261 22645456 PMC3356741

[52] Ishii T, Asuwa N. Collagen and elastin degradation by matrix metalloproteinases and tissue inhibitors of matrix metalloproteinase in aortic dissection. Hum Pathol. 2000;31: 640–646. 10.1053/hupa.2000.7642 10872655

[53] Zhang X, Wu D, Choi JC, Minard CG, Hou X, Coselli JS, et al Matrix metalloproteinase levels in chronic thoracic aortic dissection. J Surg Res. 2014;189: 348–358. 10.1016/j.jss.2014.03.027 24746253 PMC4065027

[54] Koullias GJ, Ravichandran P, Korkolis DP, Rimm DL, Elefteriades JA. Increased Tissue Microarray Matrix Metalloproteinase Expression Favors Proteolysis in Thoracic Aortic Aneurysms and Dissections. Ann Thorac Surg. Elsevier; 2004;78: 2106–2110. 10.1016/j.athoracsur.2004.05.088 15561045

[55] Ejiri J, Hirata K, Yokoyama M, Kawashima S, Inoue N, Munezane T, et al Oxidative stress in the pathogenesis of thoracic aortic aneurysm: Protective role of statin and angiotensin II type 1 receptor blocker. Cardiovasc Res. 2003;59: 988–996. 10.1016/S0008-6363(03)00523-6 14553839

[56] Arcucci A, Ruocco MR, Albano F, Granato G, Romano V, Corso G, et al Analysis of extracellular superoxide dismutase and Akt in ascending aortic aneurysm with tricuspid or bicuspid aortic valve. Eur J Histochem. PAGEPress Publications, Pavia, Italy; 2014;58: 2383 10.4081/ejh.2014.2383 25308842 PMC4194390

[57] Billaud M, Phillippi JA, Kotlarczyk MP, Hill JC, Ellis BW, St Croix CM, et al Elevated oxidative stress in the aortic media of patients with bicuspid aortic valve. J Thorac Cardiovasc Surg. 2017/05/25 ed. 2017;154: 1756–1762. 10.1016/j.jtcvs.2017.05.065 28651938 PMC5992908

[58] Phillippi JA, Klyachko EA, Kenny 4th JP, Eskay MA, Gorman RC, Gleason TG. Basal and oxidative stress-induced expression of metallothionein is decreased in ascending aortic aneurysms of bicuspid aortic valve patients. Circulation. 2009/04/27 ed. 2009;119: 2498–2506. 10.1161/CIRCULATIONAHA.108.770776 19398671 PMC5268483

