# Supplementary material for: Extracellular matrix in ascending aortic aneurysms and dissections – What we learn from decellularization and scanning electron microscopy
Source: PLoS One. 2019 Mar 18;14(3):e0213794. doi: 10.1371/journal.pone.0213794 (PMC6422325; doi:10.1371/journal.pone.0213794)
Supplement: S1 File — (DOCX) [file pone.0213794.s001.docx]

**S1 – online supplement**

to

**Extracellular matrix in ascending aortic aneurysms and dissections –**

**What we learn from decellularization and scanning electron microscopy**

Short title:

Scanning electron microscopic observation of aneurysmal and dissection tissue

Teresa Mimler^1^, Clemens Nebert^1^, Eva Eichmair^1^, Birgitta Winter^1^, Thomas Aschacher^2^, Marie-Elisabeth Stelzmueller^2^, Martin Andreas^2^, Marek Ehrlich^2^, Guenther Laufer^2^, Barbara Messner B.^1*^

^1^ Department of Surgery, Cardiac Surgery Research Laboratory, Medical University of Vienna, Vienna, Austria

^2^ Department of Surgery, Cardiac Surgery, Medical University of Vienna, Vienna, Austria

***** Corresponding author

E-mail: barbara.messner@meduniwien.ac.at (BM)

**Material and methods**

Statistical analyses were performed using IBM SPSS 24.0 software. All data were first analysed for distribution (Gaussian distribution). For the comparison of mean values between native and decellularized tissue of correspondingly related groups either a t-test (pair-wise comparison, parametric) or a Wilcoxon test (non-parametric) was performed. In order to test for differences in mean values between non-aneurysmal tissue and the disease groups (aneurysms and dissections) within the two groups (native and decellularized) a t-test for independent samples (parametric data) or a Mann-Whitney-U test (non-parametric data) was performed. A p-value ≤0.05 was defined as statistically significant.

**Results**

To determine statistically significant differences between native and decellularized tissue of correspondingly related groups (e.g. non-aneurysmal native versus non-aneurysmal decellularized) a t-test or Wilcoxon test (dependent on Gaussian distribution) was performed and corresponding p-values are depicted in Table A – S1 file for nuclei count, WGA positive area, elastic fiber area, ECM area (without EF), elastin positive area and collagen area. Table B – S1 file shows the p-values after comparison of mean values of non-aneurysmal tissue with the three diseased groups (within the two treatment groups) for nuclei count, WGA positive area, elastic fiber area, ECM area (without EF), elastin positive area and collagen area using a t-test (independent samples) or a Mann-Whitney U test (for non-parametric data).

**Table A – S1 file.** **Statistical analyses and indication of p-values for nuclei count, WGA positive area, elastic fiber area, ECM area (without EF), elastin positive area and collagen area.** Test used for this analysis is either a t-test (for pair-wise comparison) or a Wilcoxon test (non-parametric). P-values indicate differences of correspondingly related samples between the two treatment groups (native versus decellularized).

|  | | | **Native** | | | |
| --- | --- | --- | --- | --- | --- | --- |
|  |  |  | **NA** | **BAV-aTAA** | **TAV-aTAA** | **AAD type A** |
| **Nuclei counts/0.1mm^2^** | **2.5% NaOH** | **NA** | 0.008 |  |  |  |
|  |  | **BAV-aTAA** |  | <0.001 |  |  |
|  |  | **TAV-aTAA** |  |  | 0.001 |  |
|  |  | **AAD type A** |  |  |  | 0.003 |
| **WGA positive area/0.1mm^2^** | **2.5% NaOH** | **NA** | 0.008 |  |  |  |
|  |  | **BAV-aTAA** |  | 0.008 |  |  |
|  |  | **TAV-aTAA** |  |  | 0.008 |  |
|  |  | **AAD type A** |  |  |  | <0.001 |
| **Elastic fiber area/1000µm^2^** | **2.5% NaOH** | **NA** | n.s. |  |  |  |
|  |  | **BAV-aTAA** |  | n.s. |  |  |
|  |  | **TAV-aTAA** |  |  | n.s. |  |
|  |  | **AAD type A** |  |  |  | n.s. |
| **ECM area (without EF)/1000µm^2^** | **2.5% NaOH** | **NA** | 0.004 |  |  |  |
|  |  | **BAV-aTAA** |  | <0.001 |  |  |
|  |  | **TAV-aTAA** |  |  | n.s. |  |
|  |  | **AAD type A** |  |  |  | n.s. |
| **Elastin positive area/1000µm^2^** | **2.5% NaOH** | **NA** | n.s. |  |  |  |
|  |  | **BAV-aTAA** |  | n.s. |  |  |
|  |  | **TAV-aTAA** |  |  | n.s. |  |
|  |  | **AAD type A** |  |  |  | n.s. |
| **Collagen area/1000µm^2^** | **2.5% NaOH** | **NA** | n.s. |  |  |  |
|  |  | **BAV-aTAA** |  | 0.024 |  |  |
|  |  | **TAV-aTAA** |  |  | 0.006 |  |
|  |  | **AAD type A** |  |  |  | n.s. |

NA = non-aneurysmal tissue, n=9; BAV-aTAA = bicuspid aortic valve associated ascending thoracic aortic aneurysm, n=9 (for elastin positive area n=8); TAV-aTAA = tricuspid aortic valve associated ascending thoracic aortic aneurysm, n=9; AAD type A = acute aortic dissection type A, n=8; WGA = wheat germ agglutinin; ECM = extracellular matrix; EF = elastic fiber; NaOH = sodium hydroxide; n.s. = not significant.

**Table B – S1 file. Statistical analyses and indication of p-values for nuclei count, WGA positive area, elastic fiber area, ECM area (without EF), elastin positive area or collagen area.** Test used for this analysis is either a t-test (independent samples) or a Mann-Whitney U test (non-parametric). P-values indicate differences between non-aneurysmal tissue and aneurysmal/dissected tissue within the two groups (native and decellularized).

|  | **Native** | | | | | **2.5% NaOH** | | | | |
| --- | --- | --- | --- | --- | --- | --- | --- | --- | --- | --- |
|  |  | **NA** | **BAV-aTAA** | **TAV-aTAA** | **AAD type A** |  | **NA** | **BAV-aTAA** | **TAV-aTAA** | **AAD type A** |
| **Nuclei counts/0.1mm^2^** | **NA** |  | n.s. | n.s. | n.s. | **NA** |  | n.s. | n.s. | n.s. |
| **WGA positive area/0.1mm^2^** | **NA** |  | n.s. | 0.047 | 0.034 | **NA** |  | n.s. | n.s. | n.s. |
| **Elastic fiber area/1000µm^2^** | **NA** |  | n.s. | n.s. | n.s. | **NA** |  | n.s. | n.s. | n.s. |
| **ECM area (without EF)/1000µm^2^** | **NA** |  | n.s. | n.s. | n.s. | **NA** |  | n.s. | n.s. | n.s. |
| **Elastin positive area/1000µm^2^** | **NA** |  | n.s. | n.s. | 0.034 | **NA** |  | n.s. | n.s. | n.s. |
| **Collagen area/1000µm^2^** | **NA** |  | n.s. | 0.006 | n.s. | **NA** |  | 0.031 | n.s. | n.s. |

NA = non-aneurysmal tissue, n=9; BAV-aTAA = bicuspid aortic valve associated ascending thoracic aortic aneurysm, n=9 (for elastin positive area n=8); TAV-aTAA = tricuspid aortic valve associated ascending thoracic aortic aneurysm, n=9; AAD type A = acute aortic dissection type A, n=8; WGA = wheat germ agglutinin; ECM = extracellular matrix; EF = elastic fiber; NaOH = sodium hydroxide; n.s. = not significant.
